# Toleration of Morphine

**Published:** 1880-05

**Authors:** 


### TOLERATION OF MORPHINE.

DR. F. POWERS, of Westport, Conn., writes that a woman about sixty years old entered a drug-store in the town a short time ago, and called for forty-five grains of morphine. The druggist weighed it out; she then called for a glass of water, put the drug in it and swallowed the whole. The anxiety of the druggist was somewhat relieved by her saying that she had taken morphine for forty-five years, and that forty-five grains was now her regular dose.—*Medical Record.*

